# c-Myc Upregulated by High Glucose Inhibits HaCaT Differentiation by S100A6 Transcriptional Activation

**DOI:** 10.3389/fendo.2021.676403

**Published:** 2021-05-14

**Authors:** Jie Zhang, Peilang Yang, Dan Liu, Min Gao, Jizhuang Wang, Xiqiao Wang, Yan Liu, Xiong Zhang

**Affiliations:** Department of Burn, Ruijin Hospital Affiliated to Shanghai Jiao Tong University School of Medicine, Shanghai, China

**Keywords:** c-Myc, S100A6, differentiation, diabetes, wound healing

## Abstract

Keratinocyte differentiation dysfunction in diabetic skin is closely related to impaired skin barrier functions. We investigated the effects of c-Myc and S100A6 on Human immortal keratinocyte line (HaCaT) or keratinocyte differentiation and potential mechanisms. The expression levels of differentiation makers such as transglutaminase 1 (TGM1), loricrin (LOR), and keratin 1 (K1) were significantly reduced, while the expression of c-Myc was significantly increased in HaCaT cells cultured in high glucose and wound margin keratinocytes from diabetic rats and human patients. Overexpression of *c-Myc* caused differentiation dysfunction of HaCaT, while knocking down *c-Myc* promoted differentiation. High glucose increased the expression of c-Myc and inhibited differentiation in HaCaT cells by activating the WNT/β-catenin pathway. Moreover, inhibition of c-Myc transcriptional activity alleviated the differentiation dysfunction caused by high glucose or overexpression of *c-Myc*. c-Myc binds to the *S100A6* promoter to directly regulate *S100A6* expression and high glucose promoted *S100A6* transcription. The expression of S100A6 was increased in HaCaT cultured with high glucose and wound margin keratinocytes from diabetic rats and human patients. However, the expression of S100A6 was decreased during normal HaCaT differentiation. HaCaT cells treated with S100A6 recombinant protein showed differentiation dysfunction. The expressions of TGM1, LOR and K1 in knockdown *S100A6* HaCaT cells were higher than those in the control group. Overexpression of *c-Myc* or high glucose caused differentiation dysfunction of HaCaT cells, and was rescued by knocking down *S100A6*. These findings illustrate a new mechanism by which c-Myc upregulated by high glucose inhibits HaCaT differentiation by directly activating *S100A6* transcription. Thus, c-Myc and S100A6 may be potential targets for the treatment of chronic diabetic wounds.

## Introduction

The skin acts as a barrier that helps the body resist external pathogenic microorganisms and maintain a stable internal environment. The epidermis forms this defence barrier during differentiation, which begins when basal keratinocytes exit the cell cycle and move through the basal, spinous, and granular layers, and the stratum corneum (1). Disordered keratinocyte differentiation is an important cause of impaired diabetic skin barrier function (2).

Previous studies have shown that diabetic mice exhibit disordered skin differentiation (3, 4). In chronic wounds, keratinocytes at the wound margin showed high proliferation without differentiation (5). In our previous studies, we also found that keratinocytes at the diabetic wound margin are characterised by keratinocyte differentiation dysfunction and that c-Myc was upregulated in diabetic wounds (unpublished results).c-Myc regulates the expression of many genes related to cell proliferation, migration, differentiation, and apoptosis (6). Activated β-catenin and c-Myc in the epidermis of chronic wounds may be molecular markers of impaired healing (7). The transgenic mouse overexpressing the *c-Myc* gene in the epidermis showed high proliferation and inhibited differentiation of keratinocytes (8). However, the specific mechanism by which c-Myc regulates differentiation of keratinocytes is still unclear. As a transcription factor, we hypothesised that c-Myc could regulate keratinocytes by transcriptionally controlling target genes. Although numerous c-Myc target genes have been found, the specific target genes of c-Myc in regulating the fate of keratinocytes are still elusive (9). Many important proteins in the process of epidermal differentiation are encoded by genes clustered on human chromosome 1q21 and these genes constitute the epidermal differentiation complex (EDC) (10). Analysis of a ChIP-seq database (Cistrome Data Browser: 8122) showed that the S100 calcium binding protein A6 (*S100A6*) was most likely the target gene of c-Myc (11, 12). The *S100A6* belongs to EDC and its protein is characterized by two EF-hand calcium-binding motifs (13). Moreover, S100A6 promotes the proliferation of endothelial cells and inhibits their differentiation (14).

Based on the literature and our previous results, we hypothesised that the epidermal differentiation dysfunction observed at the diabetic wound margin might be closely related to c-Myc and its target gene, *S100A6*. The present study aimed to investigate the effects of c-Myc and S100A6 on Human immortal keratinocyte line (HaCaT) or keratinocyte differentiation and potential mechanisms.

## Materials and Methods

### Human Wound Specimens

Diabetic wound tissue was obtained from debridement or amputation of diabetic foot patients over a course of more than three months. Non-diabetic wound tissue in the control group was obtained from debridement of acute trauma injury or plastic surgery. Written informed consent was obtained from participating patients. The procedure was approved by the ethics committee of Shanghai Jiao Tong University School of Medicine (SJTUSM) (Number:2016-105-T54).

### Cell Culture

HaCaT and 293T cells were obtained from Fu Heng Biology (Shanghai, China). Cells were cultured in defined keratinocyte-serum free medium (K-SFM, 10744–019, Gibco, Waltham, MA, USA) supplemented with high glucose (30 mM) or normal glucose (9 mM) for 5 d at 37°C in 5% CO_2_. HaCaT differentiation was induced by increasing the concentration of CaCl_2_ to 2.8 mM. Cells were pre-treated with 20 μM 10058-F4 (S7153, Selleck, Houston, TX, USA) or 25 μM KYA1797K (S8327, Selleck) or 1 μM S100A6 recombinant protein(10939-HNAE, Sino Biological Inc. Beijing, China) for 24 h. Overexpression of *c-Myc* (*c-Myc* ^OE^), knock-down *c-Myc* (*shMyc*,Target Seq : GAGGCGAACACACAACGTCTT) and knock-down *S100A6* (*shS100A6*,Target Seq: TGCAAGGCTGATGGAAGACTT) were introduced into HaCaT by transfection with lentivirus synthesised at Shanghai GeneChem Company (Shanghai, China). Stable cell clones were obtained through selection on 2 μg/ml puromycin. Cells were harvested when cell confluence reached to about 80%. Cells were collected for protein or mRNA extraction or fixed with 10% polyformaldehyde for immunofluorescence analysis.

### Animals

Thirty rats were obtained and housed at the Animal Science Center of SJTUSM. The procedure was approved by the Institutional Animal Care and Use Committee of SJTUSM. Rats were randomly divided into three groups: the control group (Ctrl, n =10), diabetic group (DM, n =10) and diabetic rats treated with an c-Myc inhibitor group (DM+10058-F4, n =10). A high-fat diet combined with streptozotocin injections was used to establish a diabetic rat model as previously described (15). Briefly, rats in the control group were fed a normal chow diet, while rats in the diabetic group were fed a high-fat diet containing 60% (kcal) fat, 20% (kcal) carbohydrates, and 20% (kcal) protein (Research diets, D12492, Research Diets Inc., New Brunswick, NJ, USA) for 8 weeks. Subsequently, the rats in the diabetic group were fasted for 16 h and then intraperitoneally injected with 10 mg/kg streptozotocin (ALX-380-010, Enzo, Farmingdale, NY, USA) dissolved in 0.1 M citrate buffer for 4 consecutive days. Rats were then allowed to develop diabetes for 4 weeks and resumed a high-fat diet. Rats in the control group received intraperitoneal injections of saline. Random blood glucose level >16.6 mM was considered as an indicator of diabetes.

### Wounding Procedure

Rats were anaesthetized with a single intraperitoneal injection of 3% sodium pentobarbital (60mg/kg) and the hair was removed from the backs of the rats. Four full-thickness skin wounds on the mid-back were created with a 9-mm punch biopsy. The wounds of rats in the control and DM groups were treated with saline, while the c-Myc inhibitor group was topically administered 30 mg/kg 10058-F4 for 11 consecutive days. Wound and an additional 5 mm of the surrounding normal skin tissues were collected on day 11 post-injury. Half of the tissues were fixed in 4% paraformaldehyde solution and the other half were frozen in liquid nitrogen. Paraffin sections of the wound tissues were stained with haematoxylin and eosin (H&E).

### Western Blotting

Protein was extracted from HaCaT cells and measured with the BCA assay. Proteins (20 μg) were separated by Biofuraw™ Precast Bis-Tris Gel (180-8008H, Tanon, Shanghai, China) and then transferred to nitrocellulose membranes. After blocking with 5% skimmed milk for 1 h, the membranes were then incubated overnight at 4°C with the following primary antibodies at the dilution of 1:1000: c-Myc (10828-1-AP, Proteintech, Rosemont, IL, USA), transglutaminase 1 (TGM1,12912-3-AP, Proteintech), loricrin (LOR, 55439-1-AP, Proteintech), keratin 1(K1,16848-1-AP, Proteintech), β-catenin (51067-2-AP, Proteintech), H3 (A2348, ABclonal, Woburn, MA, USA), S100A6 (10245-1-AP, Proteintech) and β-actin (66009-1-Ig, Proteintech). The next day, the membranes were incubated with the HRP-conjugated secondary antibody (1:5000) for 1 h at room temperature. Finally, western blot bands were visualised using electrochemical luminescence. The band density analysis was performed using Image‐Pro Plus.

### RT-qPCR

Total RNA was extracted using Trizol and cDNA was synthesised using the HiScript^®^ III RT SuperMix kit (R323-01, Vazyme, Nanjing, China). Quantitative PCR (qPCR) was performed in triplicate using the ChamQ Universal SYBR qPCR Master Mix (Q711-02, Vazyme). The primers were synthesised by Shanghai Sangon Biotech (Shanghai, China) and shown in **Table 1**. The relative gene expression was calculated using the 2^−ΔΔCT^ method.

**Table 1:** Primer sequences.

Gene	Sequence (5’−3’)
GAPDH Forward	GGGAAACTGTGGCGTGAT
GAPDH Reverse	GAGTGGGTGTCGCTGTTGA
c-Myc Forward	CGTCTCCACACATCAGCACAA
c-Myc Reverse	TGTTGGCAGCAGGATAGTCCTT
TGM1 Forward	GCACCACACAGACGAGTATGA
TGM1Reverse	GGTGATGCGATCAGAGGATTC
LOR Forward	GGAGATCAGTGCTCCTCACA
LOR Reverse	AGCAGAACTAGATGCAGCCG
Keratin 1 Forward	GGTGCTTATATGACCAAGGTGG
Keratin 1 Reverse	ATGCTGTCCAGGTCGAGACT
S100A6 Forward	GGGAGGGTGACAAGCACAC
S100A6 Reverse	AGCTTCGAGCCAATGGTGAG

### Immunofluorescence

Cells were fixed in 4% paraformaldehyde for 30 min at room temperature. Paraffin sections were deparaffinized and then antigen repaired with sodium citrate. Slides were incubated with 3% hydrogen peroxide for 20 min to eliminate endogenous peroxidases. Slides were washed with phosphate buffered saline (PBS) three times and permeabilized with 0.1% Triton X-100 for 10 min. Slides were then incubated with 10% BSA in PBS for 30 min and incubated with primary antibodies against TGM1, LOR, K1, or S100A6 at a dilution of 1:100 at 4°C overnight. The next day, cells were incubated with the donkey anti-rabbit IgG secondary antibody Alexa Fluor 568 (A10042, Invitrogen, Carlsbad, CA, USA) at the dilution of 1:500 for 1 h at room temperature and with DAPI for 5 min.

### Immunohistochemistry

The pre-processing of the slides was performed as described in the previous Immunofluorescence section. Slides were then incubated with primary antibodies against c-Myc at the dilution of 1:100 at 4°C overnight and with the biotinylated secondary antibody on the next day for 1h. Finally, slides were incubated with 3,3′-diaminobenzidine tetrahydrochloride and the reaction time was controlled under the microscope. Brown particles were considered positive.

### Chromatin Immunoprecipitation Assay (ChIP)

HaCaT cells (1×10^7^) were used for ChIP according to a standard protocol using the Enzymatic Chromatin IP Kit (9003, CST). DNA immunoprecipitated with an anti-c-Myc antibody (9402S, CST), IgG or H3 was examined by qPCR using a pair of primers that encompassed the potential c-Myc binding site in the *S100A6* promoter: Forward GCCTTCACTCCCCCGTAAA; Reverse CCTCAGTGCCCCAAATTCCA. The ChIP enrichment efficiency was calculated using the following formula: percent input = 2% × 2^(CT 2%Input Sample–CT IP Sample)^. Finally, the qPCR products were separated with 1.5% agarose gel electrophoresis and imaged under ultraviolet light.

### Dual Luciferase Reporter Assay

pMyc-TA-luc vector was obtained from Beyotime Biotechnology (D2198, Shanghai, China). A 2.0-kbp promoter region of *S100A6* (S100A6-WT) or with mutation in the potential c-Myc binding site of (S100A6-Mut) was cloned into a pGL3-Basic luciferase reporter vector (Umibio Co. Ltd, Shanghai, China). The *c-Myc* overexpression plasmid and its control plasmid were obtained from the Shanghai GeneChem Company. *S100A6* promoter activity was normalised by co-transfection with a Renilla luciferase reporter (Umibio, Shanghai, China). Luciferase activities for firefly and Renilla were measured in 293T cells with a dual-luciferase reporter kit (MA0518, Meilunbio, Dailan, China) 36 h after transfection.

### Statistical Analysis

All values are expressed as means ± SEM. Data was analysed using SPSS 24 (IBM Corp., Armonk, NY, USA). Statistical comparisons were performed using the Student’s t-test or one-way analysis of variance (ANOVA) followed by the Tukey’s post-test. P values < 0.05 were considered statistically significant.

## Results

### High Glucose or Diabetes Impairs HaCaT or Keratinocytes Differentiation

**Figure 1:**
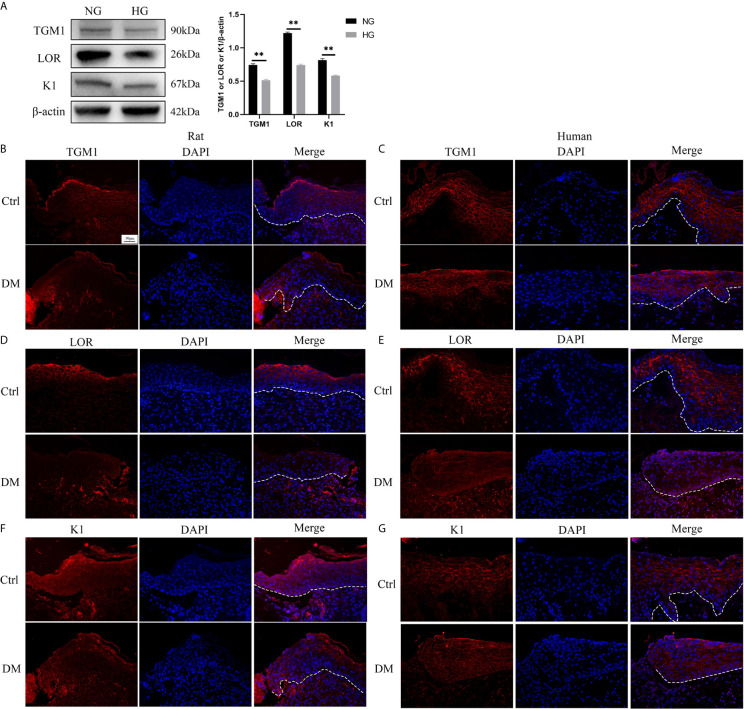
High glucose or diabetes impairs HaCaT or keratinocytes differentiation. **(A)** The expression levels of differentiation makers such as TGM1, LOR, and K1 were analyzed by western blot. **(B–G)** TGM1, LOR, and K1 expression levels were determined by immunofluorescence assay in wound margin tissues of rats **(B, D, F)** or humans **(C, E, G)**. The nuclei were stained blue using DAPI. Magnification: ×200, Scale bar = 50 μm. The white dotted line represents the epidermis-dermis dividing line. Data were shown as the mean ± SEM measured in triplicate from three independent experiments. **P < 0.01.

There are few reports on the effect of high glucose on the differentiation of HaCaT. Thus, we determined the effect of high glucose on the differentiation of HaCaT cells and keratinocytes at diabetic wound margin. The expressions of differentiation makers such as TGM1, LOR, and K1 in the high glucose (HG) group were significantly lower than those in the normal glucose (NG) group (**Figure 1A**). Similarly, the expression levels of TGM1, LOR and K1 in full-thickness skin defect wound margin tissues from diabetic rats and patients were significantly lower than those in the control group (**Figures 1B–G**). Altogether, these results indicated that high glucose or diabetes impaired HaCaT or keratinocytes differentiation.

### High Glucose Inhibits Differentiation by Increasing the Expression and Transcription Activity of c-Myc

**Figure 2:**
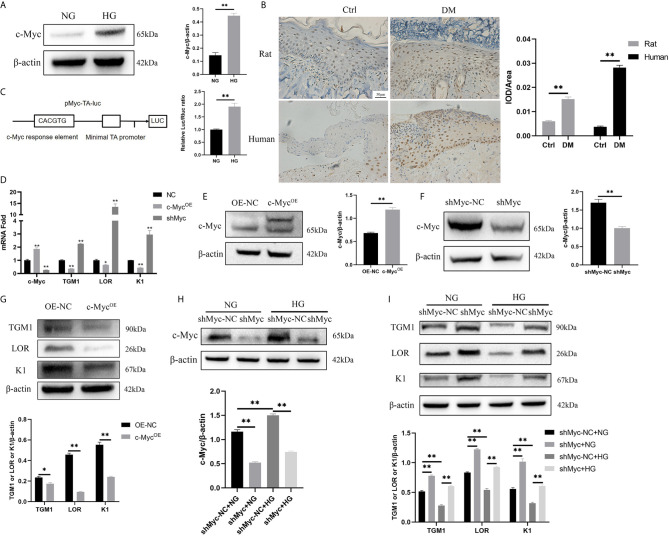
High glucose inhibits differentiation by increasing the expression and transcription activity of *c-Myc* **(A, B)** *c-Myc* expression was analyzed by western blot **(A)** and immunochemistry **(B)**. Magnification: ×200, Scale bar = 50 μl. **(C)** Schematic illustration of the *c-Myc* transactivation assay. 293T cells were cultured with high glucose or normal glucose for 5 days and then transfected with 500 ng pMyc-TA-luc and 50 ng Renilla. Firefly and Renilla luciferase activities were measured 36 h after transfection. **(D)** mRNA levels of *c-Myc*, TGM1, LOR, and K1 in HaCaT cells with *c-Myc* overexpression or knockdown were analyzed by PCR. **(E, F)** *c-Myc* protein level after *c-Myc* overexpression **(E)** or knockdown **(F)** was analyzed by western blot. **(G)** Protein levels of TGM1, LOR, and K1 in HaCaT cells with *c-Myc* overexpression were analyzed by western blot. **(H, I)** Protein levels of *c-Myc* **(H)**, TGM1, LOR, and K1 **(I)** in HaCaT cells with *c-Myc* knockdown in the NG or HG condition were analyzed by western blot. OE-NC, the negative control of c-Myc overexpression. shMyc-NC, the negative control of *c-Myc* knockdown. Data were shown as the mean ± SEM measured in triplicate from three independent experiments. ANOVA followed by Tukey’s multiple comparisons test were performed to analyze the differences. *P < 0.05, **P < 0.01.

c-Myc is a transcription factor encompassing a helix-loop-helix structure that forms heterodimers with Max. c-Myc regulates a variety of fundamental cell biological processes including proliferation, apoptosis, differentiation, and metabolism (16). Firstly, the expression of c-Myc in HaCaT cells cultured in high glucose and keratinocytes at diabetic wound margin was observed. The expression of c-Myc in the HG condition was higher than that in the NG condition (**Figure 2A**). Similarly, c-Myc was upregulated in the wound margin of diabetic patients and rats (**Figure 2B**). pMyc-TA-luc has multiple c-Myc binding sites (E-box DNA binding element) inserted into the multiple cloning sites of pGL6-TA plasmid, which can be used to detect c-Myc transcriptional activity (17). The transcriptional activity of c-Myc in the HG condition was higher than that in the NG condition (**Figure 2C**). To assess whether c-Myc affects HaCaT differentiation, we constructed HaCaT cells with stable c-Myc overexpression (**Figures 2D, E**) or knock-down (**Figures 2D, F**). The mRNA (**Figure 2D**) and protein (**Figures 2G, I**) levels of differentiation-related genes (*TGM1*, *LOR* and *K1*) in HaCaT overexpressing *c-Myc* were significantly decreased, while those in knock-down *c-Myc* were significantly increased. Furthermore, knockdown *c-Myc* (**Figure 2H**) could reverse the decrease of TGM1, LOR and K1 protein levels caused by high glucose (**Figure 2I**). These results indicated that overexpression of *c-Myc* caused differentiation dysfunction, while knocking down *c-Myc* promoted differentiation.

### High Glucose Causes Increased c-Myc by Activating the WNT/β-Catenin Pathway

**Figure 3:**
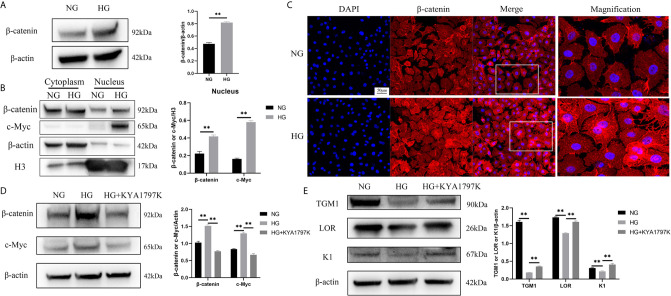
High glucose causes increased c-Myc by activating the WNT/β-catenin pathway **(A)** Total β-catenin expression was analyzed by western blot. **(B)** The expressions of β-catenin and c-Myc in the cytoplasm and nucleus were analyzed by western blot. **(C)** The expression of β-catenin was determined by immunofluorescence assay. The nuclei were stained blue using DAPI. Magnification: ×200, Scale bar = 50 μm. **(D, E)** HaCaT cells were pre-treated with Wnt/β-catenin inhibitor (KYA1797K, 25 μM) for 24 h, and then expressions of β-catenin, c-Myc **(D)**, TGM1, LOR, and K1 **(E)** were analyzed by western blot. Data were shown as the mean ± SEM measured in triplicate from three independent experiments. ANOVA followed by Tukey’s multiple comparisons test were performed to analyze the differences. **P < 0.01.

*c-Myc* is the target gene of the WNT/β-catenin pathway (18). Therefore, we speculated that high glucose induced increased c-Myc expression through the WNT/β-catenin pathway. The total protein expression of β-catenin in the HG condition was higher than that in the NG condition (**Figure 3A**). Similarly, the expressions of β-catenin and c-Myc in the nucleus in the HG condition were remarkably up-regulated than those in the NG condition (**Figure 3B**). In addition, immunofluorescence showed up-regulated expression of β-catenin in the nucleus (**Figure 3C**). The hallmark of WNT pathway activation is the nuclear translocation of β-catenin (19). The higher expression of β-catenin in the nucleus in the HG condition could be a consequence of increased total level of β-catenin. Furthermore, the expression changes of c-Myc and differentiation indicators (TGM1, LOR and K1) were observed after inhibiting the WNT pathway of HaCaT cells. KYA1797K is a potent and highly selective Wnt/β-catenin inhibitor (20). KYA1797K abolished the increased expressions of β-catenin and c-Myc caused by high glucose (**Figure 3D**). Similarly, KYA1797K prevented the decrease in TMG1, LOR and K1 expressions caused by high glucose (**Figure 3E**). These results showed that high glucose increased the expression of c-Myc and inhibited differentiation in HaCaT cells by activating the WNT/β-catenin pathway.

### Inhibition of c-Myc Transcriptional Activity Alleviates Differentiation Dysfunction Caused by Overexpression of *c-Myc* or High Glucose

**Figure 4:**
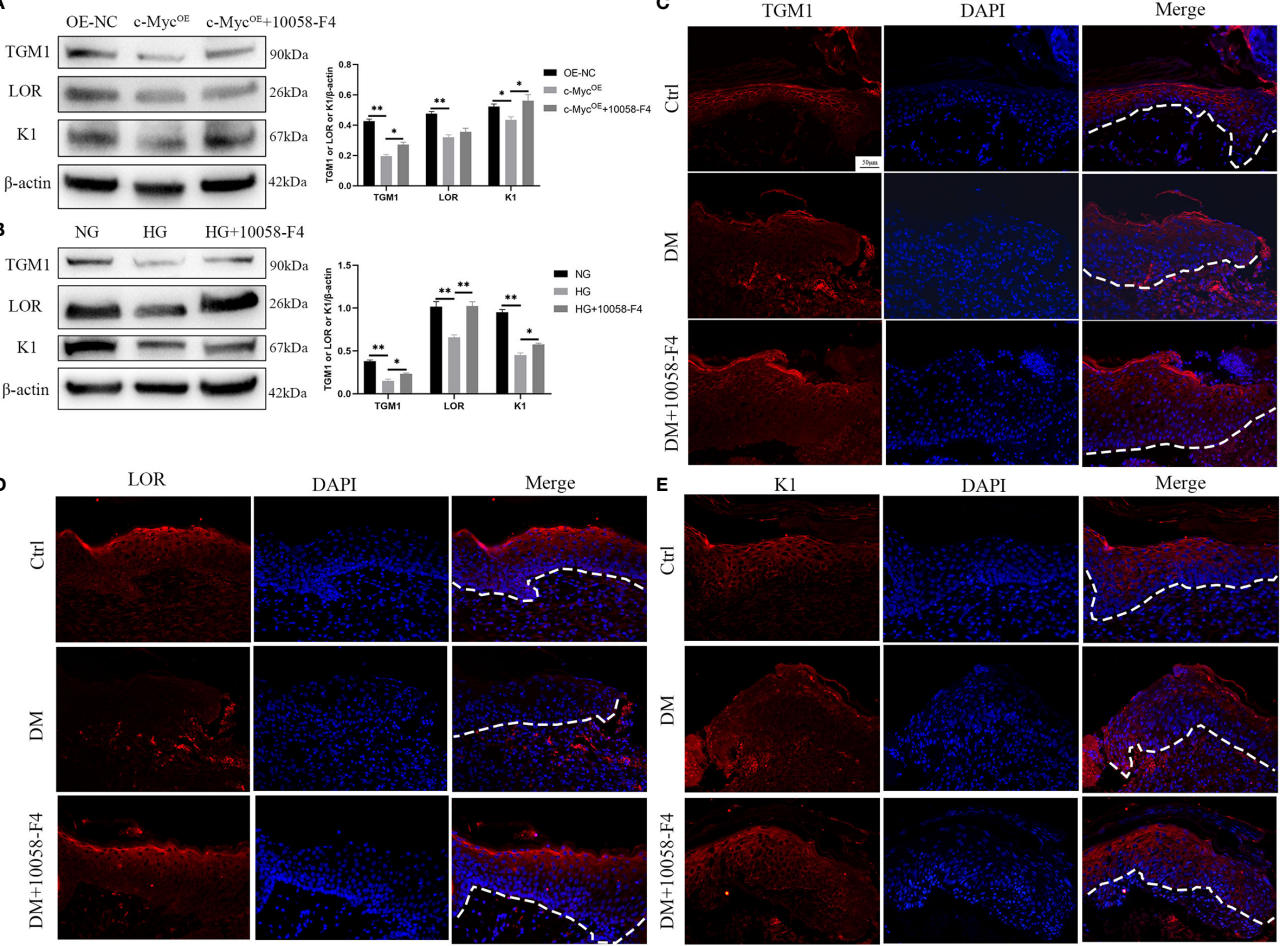
Inhibition of c-Myc transcription activity alleviates the differentiation dysfunction caused by overexpression of *c-Myc* or high glucose **(A, B)** HaCaT cells were pre-treated with c-Myc transcription activity inhibitor (10058-F4, 20 μM) for 24 h, and then expressions of TGM1, LOR, and K1 were analyzed by western blot. **(C–E)** The wounds in the Ctrl and DM groups were treated with saline, while those in the DM+10058-F4 group were topically treated with 10058-F4 (30 mg/kg) every day for 11 consecutive days. Expressions of TGM1 **(C)**, LOR **(D)**, and K1 **(E)** were determined by immunofluorescence assay in day 11 wound margin tissues of rats. The nuclei were stained blue using DAPI. Magnification: ×200, Scale bar = 50 μm. The white dotted line represents the epidermis-dermis dividing line. OE-NC, the negative control of *c-Myc* overexpression. Data were shown as the mean ± SEM measured in triplicate from three independent experiments. ANOVA followed by Tukey’s multiple comparisons test were performed to analyze the differences. *P < 0.05, **P < 0.01.

We speculated whether the inhibition of differentiation by c-Myc was related to its function as a transcriptional regulator. 10058-F4 specifically inhibits the c-Myc-Max interaction and prevents transcriptional activation of c-Myc target genes (21). Overexpression of *c-Myc* in HaCaT cells resulted in the decrease of TGM1 and K1 protein levels, which was prevented by the addition of the c-Myc inhibitor 10058-F4. The expression of LOR was significantly decreased in HaCaT overexpressing *c-Myc.* Although the LOR expression of the overexpressing *c-Myc* cells treated with 10058-F4 showed an upward trend, there was no statistical difference (**Figure 4A**). Similarly, 10058-F4 prevented the decrease of TGM1, LOR and K1 caused by high glucose (**Figure 4B**). Topical application of 10058-F4 to the wounds of diabetic rats (DM+10058-F4) also prevented the decrease of TGM1 (**Figure 4C**), LOR (**Figure 4D**) and K1 (**Figure 4E**) in keratinocytes at the wound margin as shown in the DM group. Taken together, these results indicated inhibition of c-Myc transcriptional activity alleviated differentiation dysfunction caused by overexpression of *c-Myc* or high glucose.

### *S100A6* Is Directly Transcriptionally Regulated by c-Myc

**Figure 5:**
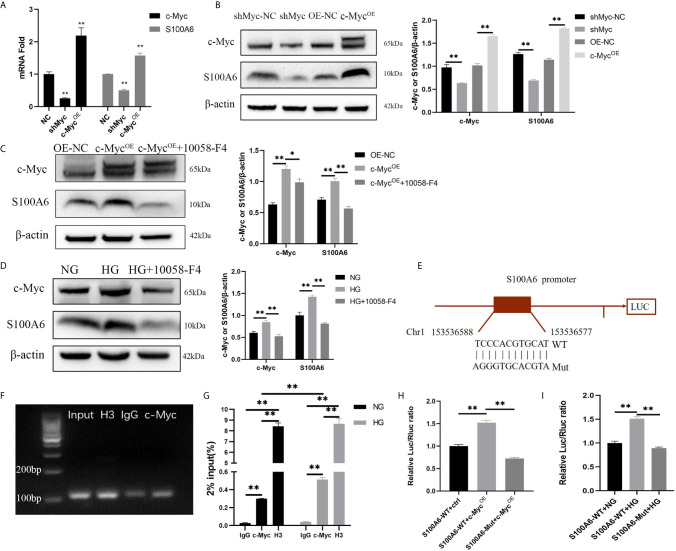
S100A6 is directly transcriptionally regulated by c-Myc **(A, B)** The mRNA **(A)** and protein **(B)** expressions of c-Myc and S100A6 were determined in HaCaT cells with *c-Myc* overexpression or knockdown. **(C, D)** The protein expression levels of *c-Myc* and *S100A6* were determined in HaCaT cells pre-treated with 10058-F4 (20 μM). **(E)** The predicted binding site of *c-Myc* in the *S100A6* promoter region on the Jaspar database (http://jaspar.genereg.net/)(relative profile score threshold 90%) and scheme of mutation strategies in the *S100A6* promoter. WT, wild-type; Mut, mutant. **(F)** ChIP-qPCR products were visualized by agarose gel electrophoresis. **(G)** The efficiency of ChIP was calculated as percent input = 2% × 2^(CT 2%Input Sample–CT IP Sample)^. **(H)** 500ng c-Myc overexpression plasmid or control plasmid was co-transfected with 500 ng plasmids containing *S100A6* gene promoter/Firefly luciferase construct and 50 ng Renilla into 293T cells in the NG condition. Firefly and Renilla luciferase activities were measured 36 h after transfection. **(I)** 293T cells were cultured in the NG or HG condition for 5d and then co-transfected with plasmids containing *S100A6* gene promoter/Firefly luciferase construct and 50 ng Renilla. Firefly and Renilla luciferase activities were measured 36 h after transfection. OE-NC, the negative control of *c-Myc* overexpression. shMyc-NC, the negative control of *c-Myc* knockdown. Data were shown as the mean ± SEM measured in triplicate from three independent experiments. ANOVA followed by Tukey’s multiple comparisons test were performed to analyze the differences. *P < 0.05, **P < 0.01.

Since the differentiation process is related to c-Myc transcriptional regulation, it is critical to identify the specific target gene of c-Myc that is involved in regulating differentiation. Firstly, we found in a ChIP-seq database (Cistrome Data Browser: 8122) that *S100A6* was most likely to be the target gene of c-Myc (12). qPCR, Western blotting, ChIP and dual luciferase reporter assay were designed to verify that *S100A6* was the target gene of c-Myc. We found that both the mRNA and protein levels of S100A6 were significantly increased in HaCaT overexpressing *c-Myc*, and significantly reduced in HaCaT with knocked-down *c-Myc* (**Figures 5A, B**). Overexpression of *c-Myc* or high glucose caused a significant increase in the S100A6 protein level compared with the control and could be abolished by 10058-F4 treatment (**Figures 5C, D**). We predicted c-Myc binding sites in the *S100A6* promoter region with Jaspar (http://jaspar.genereg.net/). A 2.0-kbp promoter region of *S100A6* (S100A6-WT) or with mutation in the potential c-Myc binding site of (S100A6-Mut) was cloned into a pGL3-Basic luciferase reporter vector (**Figure 5E**). ChIP-qPCR products were visualized by agarose gel electrophoresis and the result demonstrated that c-Myc directly bound to the *S100A6* promotor (**Figure 5F**). Higher amounts of *S100A6* promoter DNA in the HG condition were pulled down by the anti-c-Myc antibody than those in the NG condition (**Figure 5G**). Dual-luciferase assay shown increased fluorescence ratios in the S100A6-WT+c-Myc^OE^ group or S100A6-WT+HG group and decreased fluorescence ratios in the S100A6-Mut+c-Myc^OE^ group or S100A6-Mut+HG group, respectively (**Figures 5H, I**). These results indicated that *S100A6* was directly transcriptionally regulated by c-Myc and high glucose promoted *S100A6* transcription.

### S100A6 Inhibits HaCaT Differentiation

**Figure 6:**
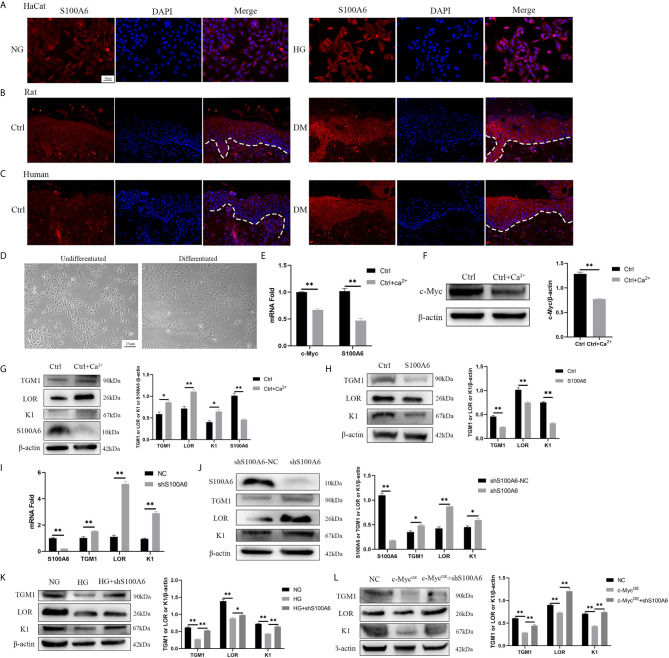
*S100A6* inhibits HaCaT differentiation **(A–C)** The expression of S100A6 was determined by immunofluorescence assay in HaCaT cells **(A)** or wound margin tissues of rats **(B)** and humans **(C)**. The nuclei were stained blue using DAPI. Magnification: ×200, Scale bar =50 μm. The white dotted line represents the epidermis-dermis dividing line. **(D)** Morphology of undifferentiated and differentiated HaCaT cells was recorded. Magnification: ×100, Scale bar =25 μm. **(E–G)** The mRNA and protein expressions of *c-Myc* and S100A6 and protein expressions of TGM1, LOR and K1 were detected after the differentiation of HaCaT cells. **(H)** HaCaT cells was pre-treated with *S100A6* recombinant protein (1 μM) for 24h and then TGM1, LOR and K1 protein levels were measured. **(I, J)** The mRNA and protein levels of TGM1, LOR, and K1 were determined after knocking down *S100A6* in HaCaT cells. **(K)** TGM1, LOR, and K1 expression levels were determined in *S100A6*-knockdown HaCaT cells cultured with high glucose. **(L)** TGM1, LOR, and K1 expression levels were determined in HaCaT cells overexpressing *c-Myc* or meanwhile knocking down *S100A6*. shS100A6-NC, the negative control of *S100A6* knockdown. NC, negative control. Data were shown as the mean ± SEM measured in triplicate from three independent experiments. ANOVA followed by Tukey’s multiple comparisons test were performed to analyze the differences. *P < 0.05, **P < 0.01.

Finally, we examined the expression of S100A6 and its effect on differentiation. The expression of S100A6 was increased in HaCaT cells cultured with high glucose (**Figure 6A**) and in keratinocytes at the wound margin from diabetic rats (**Figure 6B**) or humans (**Figure 6C**). Morphologically, undifferentiated HaCaT cells exhibited a more spindle-like shape with a more loosely connected phenotype while differentiated HaCaT cells shown a more cuboidal appearance with tightly close packing cell–cell tight junctions (**Figure 6D**). The mRNA (**Figure 6E**) and protein (**Figures 6F, G**) expressions of c-Myc and S100A6 were decreased in differentiated HaCaT cells compared with those of undifferentiated HaCaT cells. HaCaT cells pre-treated with S100A6 recombinant protein showed decreased protein levels of TGM1, LOR and K1 (**Figure 6H**). The mRNA and protein levels of *TGM1*, *LOR* and *K1* in knock-down *S100A6* cells were higher than those in the control group (**Figures 6I, J**). High glucose or overexpression of *c-Myc* caused the decrease of TGM1, LOR and K1 protein levels, which was prevented by knocking down *S100A6* (**Figures 6K, L**). These results suggested that S100A6 was highly expressed in HaCaT cells cultured with high glucose and diabetic wound margin, thereby inhibiting differentiation.

## Discussion

Skin keratinocytes undergo a complex differentiation process with multiple steps, but the exact mechanism of this process is not fully understood. Keratinocytes in the basal layer of the skin migrate upward and gradually become mitotically inactive. TGM1, LOR, and K1 are typical markers of the differentiation of keratinocytes (22). HaCaT has similar proliferation and differentiation characteristics to epidermal stem cells and HaCaT is considered a reliable differentiation model for *in vitro* studies (23, 24). Therefore, we used HaCaT cells for *in vitro* differentiation research.

Our results show that keratinocyte or HaCaT differentiation was impaired in diabetic or high glucose conditions. We explored the mechanism of epidermal differentiation dysfunction in diabetes or high glucose conditions. We observed c-Myc upregulation in keratinocytes at the margin of diabetic wounds and HaCaT cultured with high glucose, indicating that c-Myc might be related to keratinocyte or HaCaT differentiation dysfunction. we constructed HaCaT cells with stable *c-Myc* overexpression or knock-down and confirmed that overexpression of *c-Myc* caused differentiation dysfunction, while knocking down *c-Myc* promoted differentiation. We also observed that the WNT/β-catenin pathway was activated: the total protein and nucleoprotein expressions of β-catenin in the HG condition were both higher than those in the NG condition. The same phenomenon was observed in rat kidney cells cultured with high glucose (25). After the WNT pathway is activated, β-catenin enters the cell nucleus and combines with the transcription factor TCF/LEFs to initiate the transcriptional regulation of multiple target genes, including *c-Myc* (26). The mechanism by which high glucose or diabetes activates the WNT/β-catenin pathway varies in different cells. Increased oxidative stress and nitrosative stress in diabetic nephropathy rats could activate Wnt/β-catenin signalling pathway (27). High glucose induced activation of Wnt/β-catenin pathway in hepatocellular carcinoma cells by inhibiting dickkopf 4 (DKK4, a Wnt antagonist) (28). We found KYA1797K (a Wnt/β-catenin inhibitor) abolished the increased c-Myc or decreased TMG1, LOR and K1 caused by high glucose, indicating that high glucose induced increased c-Myc and differentiation dysfunction in HaCaT cells by activating the WNT/β-catenin pathway. The protein expression of c-Myc is not only regulated by upstream signal pathways, but also affected by O-GlcNAcylation (a post-translational modification) (29). Increased flux through the hexosamine biosynthetic pathway caused by high glucose induces increased UDP-GlcNAc synthesis, which is a substrate for protein O-GlcNAcylation (30, 31). It has been confirmed that c-Myc protein is more stable after being modified by O-GlcNAc in prostate cancer cells (32). In addition, our results demonstrated that high glucose caused increased transcriptional activity of c-Myc. c-Myc is O-GlcNAcylated at the site of N-terminal transcription activation domain, which suggests that O-GlcNAcylation is closely related to the transcriptional function of c-Myc (33).

It was reported that c-Myc could competitively bind Max with Mad to promote proliferation and inhibit differentiation of intestinal epithelial cells (34). Moreover, siRNA-mediated loss of *c-Myc* expression promoted human keratinocyte differentiation by down-regulating the “proliferation network” and up-regulating the “migration/adhesion-related network” (35). c-Myc-positive keratinocytes at the wound margin indicated that the wound could not heal and further debridement is needed (36). However, the specific mechanism of c-Myc regulating differentiation in the epidermis is still unknown. We wondered whether the inhibition of differentiation by c-Myc was related to its function as a transcriptional regulator. 10058-F4 is a c-Myc inhibitor that specifically inhibits the c-Myc-Max interaction and prevents transcriptional activation of c-Myc target genes (21). In addition, 10058-F4 is reported to inhibit the expression of c-Myc protein in a dose-dependent manner (37, 38). We found that inhibition of c-Myc transcriptional activity by 10058-F4 alleviated the differentiation dysfunction caused by high glucose or diabetes. We found that the expressions of S100A6 mRNA and protein were concomitantly affected by changes in *c-Myc* expression. Furthermore, overexpression of *c-Myc* or high glucose caused a significant increase in S100A6 protein levels, which was abolished by 10058-F4 treatment. These results suggest that *S100A6* is a target gene of c-Myc. Furthermore, the ChIP and dual luciferase reporter experiments confirmed that c-Myc could bind to the *S100A6* promoter region and directly transcriptionally regulate *S100A6*. A previous study reported that c-Myc binds DNA preferentially to the E-box sequence (CACGTG) (39). Indeed, The *S100A6* promoter region bound by c-Myc (TCCCACGTGCAT) contains the E-box motif.

We first confirmed that c-Myc could bind to the *S100A6* promoter region to transcriptionally regulate the expression of *S100A6*, which belongs to the EDC and participates in the regulation of various cellular functions, including proliferation, apoptosis, differentiation, cytoskeletal dynamics, and stress responses (14). The structure of S100A6 contains two EF hand motifs for binding of Ca^2+^. The conformational of S100A6 changes after binding of Ca^2+^, which promotes the interaction between S100A6 and the target protein (40). S100A6 is highly expressed in fibroblasts, epithelial cells, and different cancer cells. S100A6 is not only expressed in cells, but also detected in pancreatic juice and amniotic fluid, which indicates that S100A6 has extracellular effects (14, 41). S100A6 could bind to the receptor for advanced glycation end products and induce neuronal apoptosis by activating ROS-dependent JNK/caspase 3 and 7 pathway (42). S100A6 could also bind to integrin β1 to regulate the proliferation and adhesion of mesenchymal stem cells (43). Elevated *S100A6* levels have been detected in many tumours and are closely related to poor differentiation (13). The expression of S100A6 was increased in HaCaT cells cultured with high glucose and diabetic rats and human wounds. However, we also investigated the role of S100A6 in HaCaT differentiation and found that both mRNA and protein levels of S100A6 were decreased during the differentiation process. Treating HaCaT cells with recombinant protein S100A6 could inhibit differentiation, while knocking down *S100A6* could promote differentiation. HaCaT differentiation dysfunction caused by the overexpression of *c-Myc*, or high glucose was prevented by knocking down *S100A6*. These results indicated that S100A6 could inhibit HaCaT differentiation. Organotypic culture also confirmed that keratinocytes overexpressing *S100A6* were poorly differentiated, while keratinocytes with knocked-down *S100A6* were well differentiated (44). Keratinocytes overexpressing *S100A6* showed high proliferation, high adhesion, and suppressed *LOR* expression (44). Furthermore, S100A6 promotes proliferation by activating the EGFR pathway and its downstream signals in HaCaT (45). These biological effects of S100A6 favour an undifferentiated cell phenotype (44). p63 is believed to play a decisive role in epidermal development and differentiation (46) and is reported to transactivate the promoter region TGM1, K1 (47) and LOR (48). It has been reported that S100A6 could bind to p53(homologous to p63) and affect its biological effects (49). *In vitro* experiments confirmed that S100A6 could bind to p63 in a calcium-dependent manner and might have an important impact on the biological effects of p63 (50). However, it remains to be determined whether S100A6 binds to p63 and affects the regulation of p63 on its downstream target genes (TGM1, K1 and LOR).

**Figure 7:**
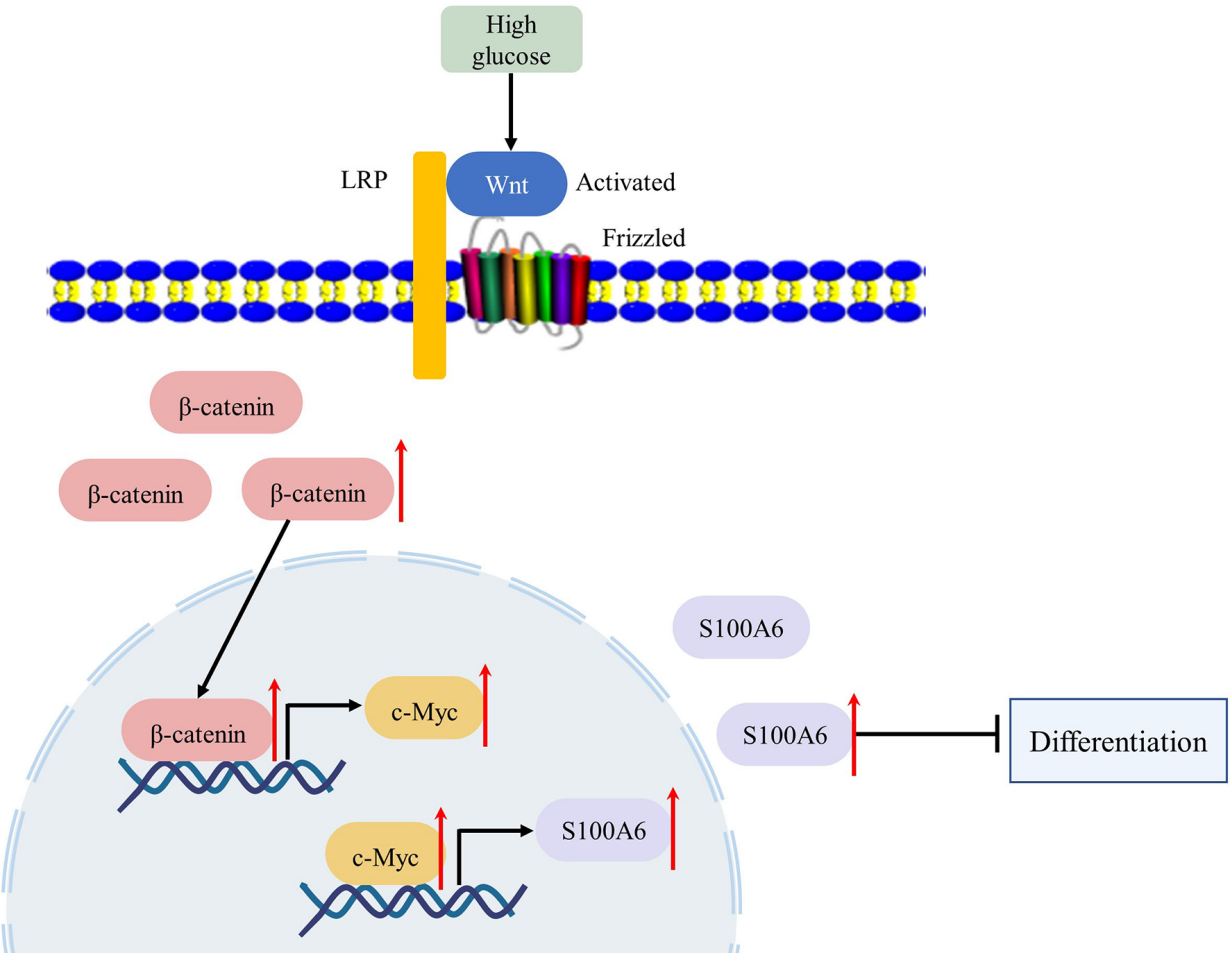
High glucose condition can increase the expression of *c-Myc* by activating the WNT/β-catenin pathway, which in turn initiates the transcription of c-Myc target gene *S100A6*, causing keratinocyte differentiation dysfunction.

In conclusion, we found that in the pathophysiological state of diabetes or high glucose, the Wnt/β-catenin signalling pathway was activated and the expression and transcription activity of c-Myc was increased. Upregulated c-Myc inhibited the differentiation of HaCaT or keratinocytes through direct transcriptional regulation of *S100A6* (**Figure 7**). Thus, c-Myc and S100A6 may be potential targets for the treatment of chronic diabetic wounds.

## Data Availability Statement

The original contributions presented in the study are included in the article/supplementary material. Further inquiries can be directed to the corresponding authors.

## Ethics Statement

The studies involving human participants were reviewed and approved by the ethics committee of Shanghai Jiao Tong University School of Medicine. The patients/participants provided their written informed consent to participate in this study. The animal study was reviewed and approved by Institutional Animal Care and Use Committee of Shanghai Jiao Tong University School of Medicine.

## Author Contributions

JZ conducted experiments and wrote the manuscript. PY and DL assisted in experiments and statistical analysis. MG and JW participated in animal experiments. XW and YL conducted project design and experimental guidance. XZ provided technical and material support and revised the manuscript. All authors contributed to the article and approved the submitted version.

## Funding

This work was supported by National Natural Science Foundation of China (No. 81871564, 82072173, and 81671914) and Shanghai Municipal Key Clinical Specialty (shslczdzk02302).

## Conflict of Interest

The authors declare that the research was conducted in the absence of any commercial or financial relationships that could be construed as a potential conflict of interest.
